# Prevalence of nonvitamin, nonmineral supplement usage among students in a Turkish university

**DOI:** 10.1186/1471-2458-5-47

**Published:** 2005-05-16

**Authors:** Unal Ayranci, Nazan Son, Osman Son

**Affiliations:** 1 Medico Social Center, Osmangazi University 26480 Meselik Eskisehir Turkey; 2 Medical Faculty, Dietitian Division, Osmangazi University 26480 Meselik Eskisehir Turkey; 3 Medical Faculty, Internal illnesses Department, Osmangazi University 26480 Meselik Eskisehir Turkey

## Abstract

**Background:**

There have been multiple studies carried out in many countries with regard to the use of nonvitamin, nonmineral (NVNM) supplements. These studies have shown that the use of NVNM supplements is on the increase throughout the world, particularly in western countries. The aim of this study was to assess the extent of NVNM supplement use among Turkish university students.

**Methods:**

The survey was conducted between September and December 2004 at Osmangazi University, a public university located in the west of Turkey. Responses were analysed, using the chi-square (x^2^) test, *t *test and percent (%) ratios, according to gender and consumers. Differences were considered significant for p ≤ 0.05.

**Results:**

Of 2253 students attending the university, 1871 participated in the survey (909 men and 962 women). Overall, the prevalence of NVNM supplement use was 16.5% (16.6% in men and 16.3% in women, p < 0.05).

The three most commonly given reasons for use were 'improvement of energy and vitality (78.6%)', 'promotion of weight loss (71.1%)', followed by 'enhancement of athletic performance (64.3%)'. Twenty-six of the 308 reported NVNM users (26/308, 8.4%) reported having experienced an adverse reaction. Television (76.3%), magazines/newspapers (41.5%) and internet websites (37.3%) were the most frequently used sources for obtaining information about NVNM supplements. The three most frequently used NVNM supplements were echinacea, ginseng, and gingko biloba (38.6%, 36.4%, and 32.8%, respectively). Nutritional scores were higher in NVNM supplement users than in non-users (66.510.8 vs. 62.712.7) (p < 0.001). Users and nonusers of NVNM supplements differed significantly according to sex, age, Body Mass Index (BMI) values, types of school, mother and fathers' education levels, family income, most permanent place of residence up to the time of survey, smoking status, and participating in sports.

**Conclusion:**

The results indicate that the prevalence of NVNM supplement use is relatively modest among Turkish university students and more information is needed on why people use particular NVNM supplements.

## Background

Dietary supplements are defined in the United States Dietary Supplement Health and Education Act of 1994 (United States DSHEA) as any product (other than tobacco) intended to supplement the diet that bears or contains one or more of the following ingredients: a vitamin, a mineral, an herb or other botanical, an amino acid, a supplement used by man to supplement the diet by increasing the total dietary intake, or a concentrate, metabolite, constituent, extract, or combination of any ingredient described above [1].

Hankin [2] and Radimer *et al. *[3] used the term 'nonvitamin, nonmineral (NVNM) supplements' to differentiate a new class of dietary supplements. NVNM supplements include products such as chrondroitin, sulfate, kava kava, ginseng, echinacea, ginkgo biloba, garlic herbals, botanicals, protein and amino acids, as well as Brewer's yeast and shark cartilage [3-5].

Although use of NVNM supplements is increasing in popularity, patterns of use for these supplements are not well known, and what is available is not always consistent, clear, or easily accessible [3].

People have reported a variety of reasons for taking dietary supplements, including decreasing their susceptibility to health problems such as stress, colds, heart attacks, osteoporosis, neural tube defects, dental caries and cancer, as well as to increase energy [6,7]. The supplements typically can be considered as falling into three distinct categories: supplements that are believed to add nutrients to a system that owing to inadequate dietary practices might otherwise be lacking [8]; supplements that allege rapid weight loss and maintenance of the loss [9], or conversely, supplements that are believed to result in weight gain or muscle development [10]. Furthermore, the rise in popularity of freely available substances purported to enhance performance, such as androsterone (a steroid precursor), and creatine (an amino acid derivative), has also been documented [11,12]. To date, however, the taking of nutritional preparations has not consistently indicated a competitive advantage other than a possible placebo effect, or one resulting from the treatment of concurrent nutritional deficiencies [13]. Consequently, according to the United States Commission on Dietary Supplement Labels, it is important for health and nutrition professionals to become more knowledgeable about all types of dietary supplements in order to help consumers make appropriate choices [14].

Variables that have been found to have a relationship with supplement use are race or ethnicity, age, education, income and lifestyle variables such as drinking, smoking, and exercising [15,16].

There is documented evidence that the use of NVNM supplements in western society is high, and its use is increasing worldwide [17-20].

In some countries, such as Germany, many botanical products are classified as drugs, and thus are subject to quality, safety, and efficacy regulations [21]. In the United States, although it is acknowledged that some dietary supplements, particularly botanicals, are used to prevent and treat diseases, DSHEA distinguishes these products from drugs and food additives and thus they are not subject to the same regulations [1,4,5]. Use of NVNM supplements increased substantially with the passage of the DSHEA [1], which gave manufacturers greater freedom to market more products as dietary supplements and to provide information about their purported benefits in package labeling and advertising. Although the United States Food and Drug Administration (FDA) regulates additives and drugs, premarket review of dietary supplements is minimal [22].

The use of NVNM supplements has increased recently in many countries, a fact also reflected in our country. However, while the worldwide rate of consumption of these supplements is near to 50% [17-19], it is still rather low in developing countries. This may be put down to indications that those with higher education and income levels seem to be comparatively more likely to use supplements [15]. In Turkey, there is no regulation of NVNM supplements in that they are widely available over the counter from such places as pharmacies and markets without a prescription. There is no regulation or health policy on the use of NVNM supplements, and furthermore, data on the prevalence and use of NVNM supplements are limited or non-existent [23]. Finally, there is a need to understand the frequency with which people use supplements and what variables are associated with particular frequencies of use.

In general, although we consider Turkish NVNM supplement consumption to fit into the prevalence patterns for developing countries, to our knowledge, no data is available either concerning use in Turkish students or the population as a whole. For this reason, the objective of this survey was to quantify the prevalence of NVNMS usage among university students; to identify supplements consumed and rationale for usage; to identify sources of supplement information; and to relate usage to selected demographic characteristics.

## Methods

### Sampling

The survey was conducted between September 2004 and December 2004 at Osmangazi University, a public university located in the west of Turkey. All students from the schools of medicine, engineering and architecture, science and literature, economics, education, and the college of health services were invited to participate. Participation was voluntary and anonymous, and the Director of the Institution approved the survey.

### Subjects

Subjects were randomly selected with a stratified sampling method. Osmangazi University is an urban, mid-sized university with six schools, all of which were included in the survey. At least one class from the first, second, third and fourth years in each department were randomly selected to participate in the survey. The sample size for the survey was determined by multiplying at least 10% by the number of the students: the total numbers of the students in each department and in our survey, respectively, were 1097 and 203 for the school of medicine; 4212 and 561 for the school of engineering and architecture; 3556 and 403 for the school of science and literature; 1394 and 178 for the school of economics; 858 and 177 for the school of education; and 302 and 212 for the college of health services.

Students were excluded from the survey due to: unwillingness to participate (n = 186), handing in incomplete questionnaires (n = 87), and non-attendance of class (n = 109). Thus, the response rate for sample respondents was 83% (1871/2253) of 2253 subjects, with ages ranging from 17 to 28, leaving a total of 1871 students (962 women, 909 men).

### Questionnaire and interview schedules

Completion of the questionnaire was self-reporting on the part of the students. The dates on which the study would be conducted were determined in cooperation with class teachers in the schools concerned. The students completed questionnaires in the presence of a member of the research team.

#### The first section of questionnaire

This section identified consumption of NVNM supplements during the survey or the past year, previous use of dietary supplements, and the probability of students considering the use of NVNM supplements in the near future. If participants reported having used a NVNM supplements during the survey period or the year prior to the study, they were further questioned on a number of topics related to consumption: the number used; the name of the product(s), 73 of which were written on the questionnaire; frequency of use of each product; total product consumption; length of product use; the reasons for using NVNM supplements, revised from previous studies [24,25]; and general demographic information such as age, sex, cigarette smoking, body height and weight, affiliation to which school of the university, class, marital status, parents' education level, and parents' total income level.

#### The second section of questionnaire

This section included the items related to nutritional beliefs about NVNM supplements. Subjects' views about NVNM supplement use and the health benefits associated with it were used to assess the motivational factors related to supplement use in this population. Items were based on those of previous studies [24-28], and also contained the most frequently consumed food items in the Turkish population. Twenty two opinions were expressed, consisting of such items as 'vitamins and minerals can provide energy', 'maintain health', increase longevity', 'reduce stress' 'prevent colds', 'improve mental function', 'reduce the risk of chronic diseases', or 'aid in recovery from fatigue', all of which were assessed to be commonly-held assumptions connected with use of the supplements. For this section, a five-point Likert scale was used, and responses were scored from one (disagree very strongly) to five (agree very strongly). Thus, the higher score showed a stronger belief on the students' part that NVNM supplements could provide specific health benefits. Applying it to a sample of 59 randomly selected students from same schools assessed the reproductivity of the questionnaire. The students who participated in the test/retest procedure were not included in the final analyses.

### Statistical analysis

Frequency, mean, and standard deviation (SD) were calculated, and the *t *test compared scores on nutritional beliefs between users and non-users, or women and men. The chi-square test (x^2^) examined the relation between general characteristics and NVNM supplements use. The percentages of the reasons cited for using NVNM supplements arranged according to sex, were calculated by first extracting out of the total figure male and female students' numbers for NVNM supplements usage, and the significance between men and women was evaluated using cross tables. The reliability of the internal consistency of data concerning nutritional beliefs collected from the pretest was calculated by Cronbach's alpha and was found to be 0.96, indicating a high internal consistency across nutritional-belief statements. Consequently, belief statement use was used for the final survey. Statistical analyses were performed using SPSS for Windows (Version 10.0, SPSS Inc., Chicago, Illinois). The standard used for statistical significance was p ≤ 0.05. The data were analyzed using descriptive statistics including means, standard deviations, and frequency distributions.

## Results

Of the participants, 909 (48.6%) were men and 962 (51.4%) women. The average age of the participants was 19.9 years (range = 17 to 28 years). Nearly half of the students (49.9%) were between the ages of 19–20. Most (74.8%) were of normal weight. More than 50% of the students (53.9%) were from the schools of engineering and architecture, and science and literature. Most students (72.4%) were in their freshman or sophomore years. Almost 100% (97.9%) were single or divorced. The proportion of students whose mothers had an education level of secondary school and lower was 61.0%, with the figure of 35.0% reported for students' fathers. Most students' level of family income (47.0%) was low. The majority of the students reported living in villages or cities (85.1%), and 26.0% were current smokers. The percentage of those participating in sport was 15.4%, and 18.0% reported exercising more than 3 times per month. Most (74.3%) rated their health as 'good/excellent'. There were significant differences between men and women according to all the descriptive information. The more detailed general characteristics of users and non-users of NVNM supplements are shown in Table 1.

**Table 1:** General characteristics of users and non-users of nonvitamin/nonmineral supplements

	**Non-users** **n(%)** **1563(83.5)**	**Users** **n(%)** **308(16.5)**	**Total** **n(%)** **1871(100.0)**
**Sex**	x^2 ^= 5.594; p = 0.018		
Men	758(83.4)	151(16.6)	909(48.6)
Women	805(83.7)	157(16.3)	962(51.4)
**Ages**	x^2 ^= 35.732; p = 0.000		
≤ 18	358(86.7)	55(13.3)	413(22.1)
19–20	789(84.6)	144(15.4)	933(49.9)
21–22	292(85.1)	51(14.9)	343(18.3)
≥ 23	124(68.1)	58(31.9)	182(9.7)
**BMI values**	x^2 ^= 73.392; p = 0.000		
Underweight (<18.5)	199(67.7)	95(32.3)	294(15.7)
Normal weight (≥ 18.5 – ≤ 24.99)	1212(86.6)	187(13.4)	1399(74.8)
Overweight (≥ 25 – ≤ 29.9)	121(90.3)	13(9.7)	134(7.2)
Obese (≥ 30 – ≤ 39.9)	31(70.5)	13(29.5)	44(2.4)
**Type of school**	x^2 ^= 23.573; p = 0.000		
Medicine	164(79.2)	43(20.8)	207(11.1)
Engineering and architecture	536(88.4)	70(11.6)	606(32.4)
Science and literature	332(82.4)	71(17.6)	403(21.5)
Economics	204(79.7)	52(16.9)	256(13.7)
Education	144(77.0)	43(23.0)	187(10.0)
College of health services.	183(86.3)	29(13.7)	212(11.3)
**Year in school**	ns		
Freshman	570(85.6)	96(14.4)	666(35.6)
Sophomore	556(80.8)	132(19.2)	688(36.8)
Junior	168(86.2)	27(13.8)	195(10.4)
Senior	269(83.5)	53(16.5)	322(17.2)
**Marital status**	ns		
Single	1520(83.7)	296(16.3)	1816(97.1)
Married	33(82.5)	7(17.5)	40(2.1)
Divorced/widow(er)/separated	10(66.7)	5(33.3)	15(0.8)
**Mother's education level**	x^2 ^= 11.940; p = 0.008		
Primary school and lower	848(84.8)	152(15.2)	1000(53.4)
Secondary school	106(74.1)	37(25.9)	143(7.6)
High school	395(84.9)	70(15.1)	465(24.9)
College or university	214(81.4)	49(18.6)	263(14.1)
**Father's education level**	x^2 ^= 7.807; p = 0.050		
Primary school and lower	416(87.6)	59(12.4)	475(25.4)
Secondary school	149(83.2)	30(16.8)	179(9.6)
High school	537(81.7)	120(18.3)	657(35.1)
College or university	461(82.3)	99(17.7)	560(29.9)
**Family income**	x^2 ^= 22.971; p = 0.000		
Low	763(86.7)	117(13.3)	880(47.0)
Moderate	566(83.4)	113(16.6)	679(36.3)
High	234(75.0)	78(25.3)	312(16.7)
**Most permanent place of residence**	x^2 ^= 13.642; p = 0.001		
Village/town	141(79.7)	36(20.3)	177(9.5)
City	1207(85.3)	208(14.7)	1415(75.6)
Metropolis	215(77.1)	64(22.9)	279(14.9)
**Smoking status**	x^2 ^= 15.435; p = 0.000		
Nonsmoker	1093(85.7)	183(14.3)	1276(68.2)
Current smoker	390(80.1)	97(19.9)	487(26.0)
Former smoker	80(74.1)	26(25.9)	108(5.8)
**Participation in sport**	x^2 ^= 9.193; p = 0.010		
No	724(82.5)	154(17.5)	878(46.9)
Former	580(82.4)	124(17.6)	704(37.6)
Yes	259(89.6)	30(10.4)	289(15.4)
**Exercise**	x^2 ^= 12.205; p = 0.002		
Never	440(83.5)	87(16.5)	527(28.2)
Fewer than 3 times per month	862(85.6)	145(14.4)	1007(53.8)
More than 3 times per month	261(77.4)	76(22.6)	337(18.0)
**Self-reported health**	ns		
Fair/poor	400(83.2)	81(16.8)	481(25.7)
Good-excellent	1163(83.7)	227(16.3)	1390(74.3)

The duration of supplement use was cited as 'between 1 and 6 months' by 65.3%, followed by 'between 6 and 12 months' and 'more than one year' (23.1% and 11.7%, respectively). More than 50% of supplement users (53.6%) were taking just one supplement at the time of this survey, followed by those using 2 supplements (38.6%) and 3 or more (7.8%). The average number of supplement taken was 1.6 (standard deviation = 0.6). Most students (68.2%) rated the frequency of supplement use as 'once/twice a day' (37.0%) or 'once every other day' (31.2%), followed by 'once/twice a week' (20.5%) and 'once/twice a month' (11.4%). No difference was revealed between men and women by supplement use (Unshown data).

Table 2 presents reasons cited for using NVNM supplements. The four most commonly given reasons for use were 'improvement of energy and vitality' (78.6%), 'promotion of weight loss' (71.1%), followed by 'enhancement of athletic performance' (64.3%) and 'retardation the onset of aging' (63.3%). Women were more likely to be using supplements to promote weight loss, burn-up fat, prevent colds, improve memory, and relieve stress; whereas men were more likely to use supplements to enhance athletic performance, retard the onset of aging, build muscle, and improve sexual function.

**Table 2:** Reasons cited for using nonvitamin/nonmineral supplements by sex

	**Men** **n(%)** **151(49.0)**	**Women** **n(%)** **157(51.0)**	**Total** **n(%)** **308(100.0)**	**x^2^; *P *values**
Improve energy and vitality	128(49.6)	114(50.4)	242(78.6)	ns
Promote weight loss	92(42.1)	127(57.9)	219(71.1)	5.59; 0.018
Enhance athletic performance	117(59.1)	81(40.9)	198(64.3)	6.54; 0.011
Retard aging	124(63.6)	71(46.4)	195(63.3)	14.41; 0.000
Burn-up fat	55(35.1)	102(64.9)	157(50.9)	14.07; 0.000
Prevent(threat) colds(sore throat)	41(35.4)	75(64.6)	116(37.6)	9.97; 0.002
Promote skin(hair) health	41(41.8)	57(58.2)	98(31.8)	ns
Build muscle	71(74.3)	24(25.7)	95(30.8)	23.25; 0.000
Supplement inadequate diet due to nutrition deficiency	48(53.9)	41(46.1)	89(28.9)	ns
Improve memory	33(39.3)	51(60.7)	84(27.3)	3.86; 0.05
Relieve stress (improve mood)	29(37.7)	48(62.3)	77(25.0)	4.67; 0.03
Improve sexual function	55(79.7)	14(20.3)	69(22.4)	24.36; 0.000
Enhance sleep	22(52.4)	20(47.6)	42(13.6)	ns
Reduce dangers of cigarette smoking	18(52.9)	16(47.1)	34(11.1)	ns
Improve circulation	18(58.1)	13(41.9)	31(10.1)	ns
Gain weight	18(62.1)	11(37.9)	29 (9.4)	ns
Prevent illnesses such as cancer, osteoporosis, menopause, hypertension, kidney stones	9(42.9)	12(57.1)	21 (6.8)	ns

Note: Subjects could list more than one reason for nonvitamin, nonmineral supplement usage

In general, when the scores obtained from all the nutritional beliefs between men and women were taken into consideration, women agreed on the effects obtained from taking NVNM supplements more strongly than men in the categories: 'important in maintaining health', 'stress reduction', and 'enhancement sleep', whereas men agreed more strongly on 'improvement sexual function' than women (Unshown data).

Twenty-six of the 308 NVNM users (26/308, 8.4%) reported having had an adverse reaction to an NVNM supplement. Of 26, the two most adverse effects were nausea and vomiting (61.5%, 30.7%, respectively), followed by gastrointestinal disturbances (15.4%), flashing (11.5%) and liver insufficiency (7.7%), Subjects could list more than one complication cited for NVNM usage (Unshown data).

The most frequent used sources for obtaining information about supplements were television (76.3%), magazines/newspapers (41.5%) and internet websites (37.3%); followed by family (20.1%), friends (19.8%), school and teachers (16.5%), books (13.3), radio (5.8%), health food stores (0.5%) and agricultural engineers (0.3%). The rates of those citing dietetic professionals, and doctor and nurse as their sources were only 4.2% and 6.8%, respectively (Unshown data).

Of 1871, 308 students (16.5%) reported using NVNM supplements during the past year, 23.0% students indicated that they were considering NVNM use in the near future. About 10% (9.7%) reported past usage. The data revealed no difference between men and women according to supplement usage. There were also no differences between men and women in the categories 'use of NVNM during the past year', 'considering NVNM supplement use in the future', and 'past usage of NVNM supplements' (p > 0.05 for each one) (Unshown data).

Of the 1690 students reporting no use of NVNM supplements in the past, 44.3% indicated that they had not considered using supplements, 34.7% reported that they had only thought about using supplements, and 11.6% reported that they had been unaware of the presence of supplements. There was a 9.4% non-response rate (Unshown data).

Table 3 summarizes information regarding the prevalence of NVNM supplement use. The three most commonly used supplements were reported as echinacea, ginseng, and gingko biloba (38.6%, 36.4%, and 32.8%, respectively). Since all of the 73 most commonly purchased supplements in Turkey and the world were listed on the questionnaire, the students reported using no supplements other than those listed.

**Table 3:** Prevalence of use of nonvitamin, nonmineral supplements

	**Total** **n(%)** **308(100.0)**
Echinacea	119(38.6)
Ginseng	112(36.4)
Gingko biloba	101(32.8)
Protein powder and or Amino acids	89(28.9)
Fish oil	84(27.3)
Bee pollen	78(25.3)
Garlic	64(20.8)
Green Tea	61(19.8)
Bee pollen	57(18.5)
St. John's wort	51(16.5)
Ginger	47(15.2)
Creatine	45(14.6)
Aloe	27 (8.7)
Lecithin	25 (8.1)
Flax	21 (6.8)
Chestnut seed	19 (6.2)
Valerian	16 (5.2)
Burdock root	15 (4.9)
Coenzyme Q	15 (4.9)
Goldenseal	12 (3.9)
Cranberry	11 (3.6)
Guarana	9 (2.9)
Chamomile tea	8 (2.6)
Sam-E	7 (2.3)
Kava kava	7 (2.3)
Melatonin	6 (1.9)
Dong Quai	4 (1.3)
Angelica	3 (0.9)
Cayenne	2 (0.6)
Borage	2 (0.6)
Astragalus	1 (0.3)
Evening primrose	1 (0.3)

The percentages total to more than 100% because some respondents reported taking more than one NVNM supplement

Table 4 shows that NVNM supplement use correlated to nutritional beliefs. Of all the beliefs, the links between 'supplement use and recovery from fatigue' and 'supplement use and prevention/threat colds/sore throat' were the ones with which users and non-users most agreed. The lower degree of perception of any 'relationship between supplement use and improved mental health' was similar between users and non-users. In general, when all the nutritional beliefs between users and non-users were taken into consideration, users agreed on the effects obtained from taking NVNM supplements more strongly than non-users except for the categories of 'prevention/threat of colds/sore throat', 'prevention of mental illnesses', 'burning-up fat accumulating in the body', 'promotion of more weight loss', and 'reduction of the harmful effects of cigarette smoking'. Of the 22 beliefs, the score from only one item, 'burning-up fat accumulating in the body' was found to be lower in users than in the non-users.

**Table 4:** Nutritional beliefs about nonvitamin/nonmineral supplements of supplement users and non-users*

**Belief statement**	**Non-users†** **n(%)** **1563(83.5)**	**Users†** **n(%)** **308(16.5)**	***P *values‡**
Improve energy and vitality	2.8 ± 0.8	3.2 ± 0.8	0.000
Important in maintaining health	2.9 ± 0.8	3.3 ± 0.8	0.000
Provide living longer	2.7 ± 0.8	2.8 ± 0.7	0.000
Help recovery from fatigue	3.0 ± 0.8	3.3 ± 0.7	0.000
Reduce stress	2.8 ± 0.7	3.0 ± 0.8	0.012
Prevent/threat colds/sore throat	3.1 ± 0.8	3.2 ± 0.7	ns
Prevent mental illnesses	2.7 ± 0.8	2.8 ± 0.8	ns
Improve mental health	2.6 ± 0.8	2.7 ± 0.8	0.016
Reduces risk of chronic illnesses	2.8 ± 0.7	3.0 ± 0.8	0.000
Retard aging	2.7 ± 0.9	2.9 ± 0.8	0.000
Burn fat accumulating in the body	2.9 ± 0.8	2.8 ± 0.8	ns
Promote weight loss more	2.8 ± 0.7	2.9 ± 0.8	ns
Build muscle more	2.9 ± 0.8	3.1 ± 0.7	0.000
Enhance athletic performance	2.9 ± 0.8	3.1 ± 0.8	0.001
Enhance sleep	2.8 ± 0.8	3.0 ± 0.8	0.000
Improve sexual function	2.8 ± 0.8	3.0 ± 0.8	0.000
Improve circulation	2.9 ± 0.8	3.2 ± 0.7	0.000
Supplement inadequate diet due to nutrition deficiency	2.9 ± 0.8	3.2 ± 0.8	0.000
Improve memory	2.8 ± 0.8	3.0 ± 0.8	0.001
Promote skin/hair health	2.9 ± 0.8	3.1 ± 0.8	0.000
Gain weight	3.0 ± 0.8	3.1 ± 0.8	0.009
Reduce dangers of cigarette smoking	2.7 ± 0.9	2.8 ± 0.9	ns

*Values are shown as mean ± standard deviation

† A five-point Likert scale was used; disagree very strongly (one point) to agree very strongly (five points)

‡ *t *test

## Discussion

To our knowledge, this is the first survey on NVNM supplements use conducted in Turkey. This survey allowed the assessment of the prevalence of NVNM supplement use in a sample of university students from different departments.

Our results indicate that about one in six students (16.5%) consumed one or more NVNM supplement during the past year. While our finding is compatible with some other study results, it is not with others: Radimer *et al*. [3] found that between 3.3 and 12% of respondents to their survey reported using NVNM supplements in 1994 and 1995, respectively. In a survey on a 1000 non-patient student population by Perkin et al. [25], a NVNM supplement usage rate of 26.3% was observed. Eisenberg et al. [29] reported this proportion as 12.1%. Furthermore, in a survey relevant to NVNM supplement use over a 12-month period by adult members of a large health maintenance organization, an estimated 32.7% of adults used at least one NVNM supplement [30]. Newberry *et al. *[31] reported that out of 272 college students canvassed, 48.5% had taken an NVNM supplement during the previous year and in a survey in the United States, it was found that 14.5% of the adults reported having used a NVNM supplement during the previous year [32]. The aforementioned studies show that the rate of NVNM supplement usage ranges from 3.3 to 48.5%. One explanation for these differences in reported usage rates could be the variation in how questions were asked regarding time frame and the types of supplements used. A further possibility could be relevant to individuals' sociodemographic characteristics, such as race or ethnicity, age, education, income and lifestyle variables such as drinking, smoking, and exercising [15,16].

As in other populations, echinacea, ginseng, gingko biloba, and protein powder and or amino acids were reported by this population to be the most popular supplements [3,33-35]. Wingate [33] found that in 1997 the three most reportedly used supplements were garlic, ginseng, and ginkgo biloba. Similarly, the three most commonly consumed NVNM supplements reported in the Slone Survey [36], conducted from 1998 to 1999, were ginseng, ginkgo biloba extract, and allium sativum. This high use of supplements in Turkey and the rest of the world may be due to the fact that those products are prevalent in the market.

In this survey, most students reported that they were healthy. This was reflected in the rationale offered by the students for supplement usage, such as increasing energy and enhancing athletic performance.

In this survey, a positive relationship was seen to exist between exercising and NVNM supplement usage. Both behaviors may be perceived as congruent with disease prevention. NHANES III [3] also found a positive relationship between exercise and NVNM usage.

The current survey found that former smokers used NVNM supplements most, which is in line with the NHANES III survey [3]. One explanation for this is that usage could be viewed as offsetting a negative behavior [25].

In this survey, 9.7% of the students reported past usage, 16.5% currently usage and 23.0% potential for use in the future. That these findings show increasing trends in the use of supplements with a corresponding increase in time may be due to media reports or advertisements in local newspapers about provision of energy and vitality, an increase in athletic performance, the retarding of aging, and the reduction of the risk of chronic diseases such as hypertension, colon cancer, breast cancer, kidney stones, and osteoporosis [37-39]. A further explanation may be that the use of NVNM supplements increased substantially with the passage of the United States Dietary Supplement Health and Education Act of 1994 (DSHEA), which gave manufacturers greater freedom to market more products through purported benefits reported in package labeling and adverting [1].

Twenty-six of the 308 NVNM users (26/308, 8.4%) reported having experienced an adverse reaction to an NVNM supplement. This rather high rate is in line with results published by Newberry *et al. *[31], which reported a reaction rate of 6.9%. This may be due to the fact that the majority of the students did not seek nutrition information from reliable sources such as doctors, nurses or dietetic professionals. In this survey, only 11.0% of supplement users cited doctors, nurses, and dietetic professionals as sources of information. However, it is important that students get nutrition information from reliable sources and receive proper nutrition education. To put this another way, that 76% of the respondents obtained information about supplementation from television may indicate that television has enormous implications for educational and public policy initiatives, and that television should be used to get more health information about supplementation.

The nutritional beliefs that subjects questioned had concerning supplements were an important factor for their use of supplements. Supplement users agreed more strongly than non-users on the health benefits of supplement use for the majority of items. Others reported similar findings [9,26].

In this survey, NVNM supplement use was higher in men than in women, in line with Radimer *et al.*' survey [3]. However, these findings are not concordant with data reported from Malaysia (40), where the prevalence of supplement use was higher in women (14.9% vs. 9.7%, respectively). No apparent reasons were disclosed for such differences; it is possible that NVNM supplement use might be related to a desire to increase athletic performance in men [41], but further studies are needed to better assess this result.

In this survey, nutritional knowledge average scores were low in both sexes (63.3 ± 12.4 for a maximum score of 110), probably because of the absence of nutritional education in primary, secondary, high schools or university in our country.

NVNM supplement use by the students was different according to sex, age, Body Mass Index (BMI) values, school type, mother and fathers' education levels, family income, most permanent place of residence, smoking status, participating in sport, and exercise. This finding shows that the rationale for supplements use changes according to subjects' general characteristics.

Of further interest is the trend for more female students in the current sample to indicate use of significantly more supplements than males. Parallel to our survey, this finding highlights the fact that female students are more likely to be dieting to lose weight, whereas male students are more likely to be controlling their diet to gain weight and build muscle, which is compatible with some other studies [42,43].

We are well aware of the limitations of the present survey. Firstly, it was performed in a single institution, and therefore the sample may not be representative of all Turkish students. Another limitation on the data was the use of the self-reporting system employed in this survey. This may have given rise to bias.

## Conclusion

This descriptive analysis helps to provide a clearer picture of NVNM supplement use. Not only does it indicate that students are indeed using or considering using supplements as a viable means to achieve their short-term fitness and or appearance goals, but also which products are most likely to be used, and indeed which individuals are most in need of education and information concerning the use and abuse of certain dietary aids. Further research, therefore, needs to focus on how NVNM nutritional supplements use information can be applied to specific user groups.

Monitoring the use of NVNM supplements within the community or university students should be of interest because these substances may affect health or interact with other medications [44]. The respondents in this survey reported 85 cases of interactions between St John's wort and prescription medications. Dietetic professionals and physicians need to consider evaluating NVNM supplement use when assessing overall nutrient and medication use by clients or students.

In summary, our results in this sample of Turkish students indicate that the prevalence of NVNM supplement use is still relatively modest, and neither associated with a healthier lifestyle nor related to a better nutritional knowledge. The need for further research on the relationships between dietary supplements and health has also been highlighted in the 1997 report by the United States Commission on Dietary Supplement Labels [45].

## Competing interests

The author(s) declare that they have no competing interests.

## Authors' contributions

AU conceived of the study, performed its design and coordination, sequence alignment, collected the data, performed the statistical analyses and drafted the manuscript, SN and SO collected the data and entered the data to SPSS packet program, All authors read and approved the final manuscript.

## Pre-publication history

The pre-publication history for this paper can be accessed here:


